# Metabolite profiling of *Andrographis paniculata* (Burm. f.) Nees. young and mature leaves at different harvest ages using ^1^H NMR-based metabolomics approach

**DOI:** 10.1038/s41598-019-52905-z

**Published:** 2019-11-14

**Authors:** Nor Elliza Tajidin, Khozirah Shaari, Maulidiani Maulidiani, Nor Shariah Salleh, Bunga Raya Ketaren, Munirah Mohamad

**Affiliations:** 1 Faculty of Sustainable Agriculture, Universiti Malaysia Sabah, UMS Sandakan Campus, Locked Bag No. 3, 90509 Sandakan, Sabah Malaysia; 2 Department of Crop Science, Faculty of Agriculture, Universiti Putra Malaysia, 43400 UPM, Serdang, Selangor Malaysia; 3 Institute of Bioscience, Universiti Putra Malaysia, 43400 UPM, Serdang, Selangor Malaysia; 4 School of Fundamental Science, Universiti Malaysia Terengganu, 21030 Kuala, Terengganu Malaysia

**Keywords:** Biochemistry, Plant sciences

## Abstract

*Andrographis paniculata* (Burm. F.) Nees. is considered as the herb of the future due to its precious chemical compounds, andrographolide (ANDRO), neoandrographolide (NAG) and 14-deoxyandrographolide (DAG). This study aims to profile the metabolites in young and mature leaf at six different harvest ages using ^1^HNMR-based metabolomics combined with multivariate data analysis. Principal component analysis (PCA) indicated noticeable and clear discrimination between young and mature leaves. A comparison of the leaves stage indicated that young leaves were separated from mature leaves due to its larger quantity of ANDRO, NAG, DAG, glucose and sucrose. These similar metabolites are also responsible for the PCA separation into five clusters representing the harvest age at 14, 16, 18, 20, 22 weeks of leaves extract. Loading plots revealed that most of the ANDRO and NAG signals were present when the plant reached at the pre-flowering stage or 18 weeks after sowing (WAS). As a conclusion, *A. paniculata* young leaves at pre-flowering harvest age were found to be richer in ANDRO, NAG and DAG compared to mature leaves while glucose and choline increased with harvest age. Therefore, young leaves of *A. paniculata* should be harvested at 18 WAS in order to produce superior quality plant extracts for further applications by the herbal, nutraceutical and pharmaceutical industries.

## Introduction

The popularity of herbs among health consumers as an alternative medicine to alleviate diseases and health maintenance are due to its reputation of being safer and cheaper than synthetic medicines. Herbs are also easily available through cultivation practice, either in mass propagation or smaller scale planting. One of the more popular herbs among the Asian population is *Andrographis paniculata* (*A. paniculata*), also known as the ‘king of bitters’ and much used in Ayurvedic medicine. The herb has a broad range of pharmacological effects, including anti-cancer, anti-diarrheal, anti-hepatitis, anti-HIV, anti-inflammatory, anti-malarial, antioxidant, cytotoxic and hepatoprotective as well as useful against cardiovascular diseases and sexual dysfunction^1–3^.

More than 20 structurally analogous diterpenoids and 10 flavonoids have been isolated from different parts of *A. paniculata* plant^4^. The bitter taste of *A. paniculata* is mainly contributed by andrographolide (ANDRO) and its analogues such as 14-deoxyandrographolide (DAG) and neoandrographolide (NAG). According to Okhuarobo *et al*.^5^ ANDRO is the most prominent in occurrence and quantity among the chemical constituents of *A. paniculata*, widely distributed in the aerial parts and roots of this plant. Several studies confirmed that the phytochemicals of *A. paniculata* were found to be higher in leaves compared to other plant parts^6–8^.

The content of specific phytochemicals of a plant or herb is known to vary with plant age. Bioactive metabolites of the medicinal plant *Melicope ptelefolia* was reported to be higher in the young leaves rather than in the mature leaves^9^. Similar results have been reported for the young leaves of *Clinacanthus nutans* by Raya *et al*.^10^. Lawal *et al*.^11^ stated that the variability in the metabolite contents could be influenced by diverse factors, including plant harvest age or stage. Yusof *et al*.^12^ also reported that the metabolites of *A. paniculata* evaluated by Fourier Transform Infra-Red (FTIR) spectroscopy, showed clear differences in their relative concentrations when harvested at 120, 150 and 180 days after transplanting. For high-quality material production, which has a direct impact on assurance of product efficacy, determining the most appropriate age or stage for crop harvest is of utmost importance, even before postharvest processing technologies is considered. The best time to harvest a crop for specific characteristics may be different for different cultivars, geographical region, and/or climatic conditions. With respect to local cultivars of *A. paniculata*, there is a need for more information in this respect, especially with respect to the contents of the various bioactive diterpene lactones.

Metabolomics is a comprehensive tool for the quantitative and qualitative analysis of all metabolites found in an organism^13,14^. It is a holistic, non-biased, qualitative and quantitative overview of the metabolites present in an organism^9,15^. The metabolite profile at various time points can be measured using several available state-of-the-art analytical tools, all with high reproducibility and sensitivity. Nuclear magnetic resonance (NMR) spectroscopy, in combination with chemometrics, is a proven technique used in metabolite profiling of various plant species^9,11,13,15^. NMR allows simultaneous detection of diverse groups of secondary metabolites and the metabolite quantification can be performed without the need for any calibration curves^15,16^. It is a rapid and highly reproducible method that requires only a modest sample preparation method^17^. Thus, this study was conducted to discriminate young versus mature leaves and explore the changes in the metabolite profile of young leaves harvested at different harvest age or stage. Results from this study are to fill in the information gap in the potential use of *A. paniculata* as a phytomedicinal plant.

## Materials and Methods

### Weather description and seed germination

Peninsular Malaysia is equatorially located at 1° and 7° north and 99° to 105° east^18^. The climate in Malaysia can be described as typical tropical climate characterised by uniform high temperature, high humidity and abundant rainfall^19^. Two types of monsoon period influenced the climate, that is the southwest and the northeast monsoons, which occur from April to September (dry season), and October to March (wet season), respectively^20^. The present study was conducted during the wet season, starting in October 2014 until January 2015 (Table 1). The herbal crop grew well and did not show any pest or disease infestations during the whole cultivation period. All plant parts were normal and had a natural green colour.

**Table 1:** Climate data recorded at the field planting sites during the four months of study.

	2014			2015
	October	November	December	January
Average temperature (°C)	28	27	27	28
Humidity (%)	81	84	85	78
Rainfall (mm)	210.2	355.3	322.9	177.8

Temperature and humidity were expressed as monthly average values and rainfall as accumulated rain per month. Source: Malaysia Meteorological Department, Ministry of Energy, Science, Technology, Environment and Climate Change.

Seeds of *A. paniculata* (Accessions 11265-Harapan), obtained from the AgroGene Bank Laboratory, Universiti Putra Malaysia (UPM), Serdang, were scarified with sandpaper and then soaked in hot water (68 ± 2 °C) for 3 min to break their dormancy^21^. The seeds were then sown in petri dishes lined with Whatman filter paper (No. 2), premoistened with sterile distilled water. The petri dishes were sealed with parafilm and placed in a growth chamber under a controlled light source and 60–70% relative humidity. At the two-leaf stage, the seedlings were transplanted into Jiffy pellets and maintained in the nursery for eight weeks until the six- to the eight-leaf stage, after which they were field transplanted.

### Field transplanting

Field transplanting was conducted on clay loam soil at the Vegetable Unit, University Agriculture Park, UPM (2° 59.225′N, 101°42.439′E, 45 m above sea level). The seedlings (six- to the eight-leaf stage) were transplanted to a 1.2 m × 27 m raised (15–20 cm) bed, in 5 replications. Prior to transplanting, 10 ton/ha each of rice husk and chicken manure, were incorporated as soil amendments to about 15 cm topsoil on each bed and left aside for seven days before field transplanting. Each bed was covered with Agrosheet plastic mulching to control weeds. The seedlings were transplanted onto the raised beds, evenly distributed at a count of one seedling/point. There were two planting rows for each bed. The distance between the planting rows on each bed was 30 cm and between plants within the row was also 30 cm. The plots were maintained according to standard cultural practices. The crop was watered using a sprinkler irrigation system, carried out twice a day in the morning and late evening, except for rainy days. Organic-based fertiliser (8: 8: 8) at the rate of 250 kg N/ha was applied three days after transplanting.

### Plant materials and extraction

**Figure 1:**
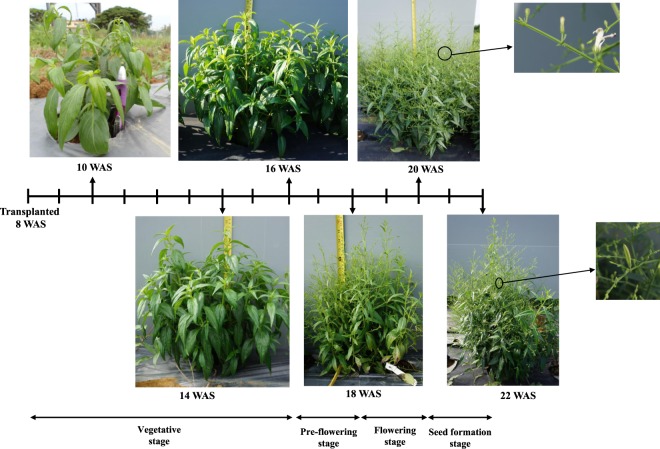
Plant harvest ages and phenological stages of *A. paniculata*.

Leaf samples from the five plots were harvested at 14, 16, 18, 20 and 22 weeks after sowing (WAS), which represent different phenological stages^22^ as describe in Fig. 1. To ensure sample uniformity, sampling was consistently carried out between 8:00 to 9:30 a.m. Six young leaves were obtained from the plant shoot tips while another six mature leaves were taken at the 5^th^ internode from the base of the plant. Upon cutting, each leaf samples were immediately quenched in liquid nitrogen and further pulverised into powder form in the cryogenic state.

The samples were then freeze-dried over 48 hours to remove residual moisture in the leaf tissues. For each dried sample, 50 mg was directly extracted with 1.5 mL of prepared deuterated solvent made up of 0.75 mL CD_3_OD and 0.75 mL KH_2_PO_4_ buffer in D_2_O (pH 6.0) containing 0.1% 3-(trimethylsilyl)propionic-2,2,3,3-d_4_ acid sodium salt (TSP), using a vortex mixer^23^. The direct extraction was further assisted by 30 min sonication in a sonicator bath (FB15055, Fisherbrand, USA), at ambient temperature. The clear deuterated supernatant obtained after centrifuging (PK110, ALC, UK) for 10 min, was transferred into NMR tubes for measurement of its ^1^H spectrum.

### ^1^H NMR spectrometry

The ^1^H NMR measurements were performed using a 500 MHz Varian INOVA NMR spectrometer (Varian Inc., California, USA) operating at 499.887 MHz, with the temperature maintained at 26 °C. Each ^1^H NMR spectrum, acquired over a spectral width of 20 ppm, consisted of 64 scans requiring 3.53 min acquisition time^11^. All spectra were manually phased, and baseline corrected using the Chenomx software (version 8.1, Alberta, Canada) it a consistent setting for all sample spectra. According to Lawal *et al*.^11^ TSP had been used as an internal standard for the chemical shift reference and the intensity scaling for all ^1^H-NMR signals. It also used as a reference in the quantitation of metabolites. The chemical shifts of all data sets were referenced to the internal standard of TSP at 0 ppm. The 2D *J*-resolved experiments were also carried out to aid metabolite identification.

### Bucketing of ^1^H NMR spectra and statistical analysis

The ^1^H NMR spectra were automatically converted to ASCII files using Chenomx software (version 8.1, Alberta, Canada). Spectral intensities were binned by equal width (δ 0.04) corresponding to the region of δ 0.50–10.00. The region δ 4.70–4.96 (water) and δ 3.28–3.33 (residual methanol) were excised from the analysis^11,13,15^. The processed and bucketed data were then subjected to multivariate data analysis. Principal component analysis (PCA) was performed with SIMCA-P software (version 13.0, Umetrics, Umeå, Sweden) using pareto scaling^9,11,13,15^^.^

One-way ANOVA (SAS version 9.4) was further conducted to determine the differences between the young and mature leaves. Leaf samples that showed higher contents of the bioactive marker compounds (e.g. ANDRO, DAG, NAD) were further evaluated based on harvest age of 14, 16, 18, 20 and 22 WAS. The means of the peak area of ^1^H NMR signals between the treatments were separated using the least significant difference (LSD) test at P ≤ 0.05. The relationships between plant age and variables (ANDRO, NAG, DAG, glucose, sucrose and choline) were determined using regression analysis. The formula to determine the plant harvest age for each optimum variable is as follows:

Regression model:

1 $$y=a+bx+c{x}^{2}$$

The equation to find the maximum parameter value:

2 $$dy/dx=b+2cx$$

where, *α*, *b* and *c* are the regression constant values and *dy*/*dx* is derivative of the y.

## Results and Discussion

### Visual inspection ^1^H NMR spectra

**Figure 2:**
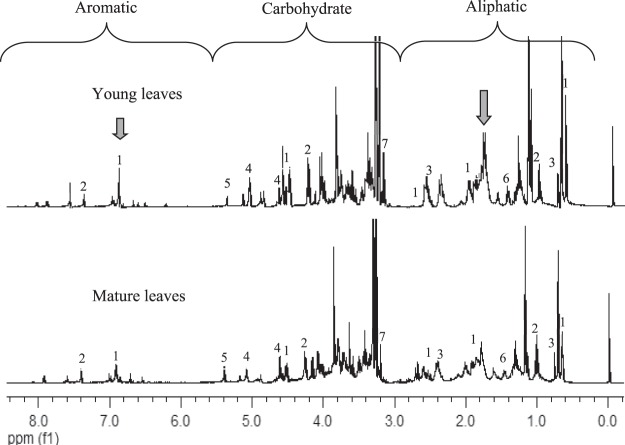
Full ^1^H NMR spectra of young and mature leaves of *A. paniculata*, from δ 0.0 to 8.0 ppm. Andrographolide (1), neoandrographolide (2), 14-deoxyandrographolide (3), glucose (4), sucrose (5), alanine (6) and choline (7).

Seven compounds were identified in the *A. paniculata* leaf extract as listed in Table 2. Identification of the compounds was carried out by matching their chemical shift values with those in the Chenomx database, NMR spectrum of the authentic standard and further supported with 2D J-resolved spectrum (see Supplementary Figs S1–S4) as well as comparison with the literature^13,23,24^. Representative ^1^H NMR spectra of the young and mature leaf extract are shown in Fig. 2. Intensely visible signals were observed in the aliphatic (δ 0.5–3.0), carbohydrate (δ 3.0–5.5), and aromatic (δ 5.5–9.0) regions, for all the spectra. Most of the signals in the aliphatic and aromatic regions were relatively more intense in the spectra of young leaves compared with those of mature leaves.

**Table 2:** Characteristic ^1^H NMR signals of compounds detected in the leaf extract of *A. paniculata*.

Metabolites	^1^H NMR characteristic signal
ANDRO	δ 6.92 (m), δ 4.88 (s), δ 4.52 (q), 2.52 (m), δ 1.92^x^, δ 0.64 (s)
NAG	δ 8.44 (s), δ 7.4 (s), 4.27 (d, *J* = 10 Hz), 1.04 (s)
DAG	δ 2.36 (d, *J* = 8.6 Hz), δ 1.16 (s), δ 0.72 (s)
Glucose	δ 5.16 (d, *J* = 3.75 Hz), δ 4.6 (d, *J* = 7.9 Hz), δ 3.84^x^, δ 3.68^x^, δ 3.24^x^
Sucrose	δ 5.4 (d, *J* = 3.6 Hz), δ 4.16 (d, *J* = 8.5 Hz)
Alanine	δ 1.44 (d, *J* = 7.9 Hz)
Choline	δ 3.20 (s)

^1^H NMR peak signals:

s, d, q, m = singlet, quartet and multiple, respectively.

^x^obscured by overlapping with other signals.

This observation is similar to those of Shuib *et al*.^9^, who reported that young leaves of *Melicope ptelefolia* showed more intense ^1^H NMR signals in the aliphatic and aromatic regions as compared to mature leaves. As shown in Table 2 and Fig. 2, the ^1^H NMR signals observed in the aliphatic and aromatic regions were those of ANDRO (δ 6.92, 4.88, 4.52, 2.52, 1.92 and 0.64), NAG (δ 8.44, 7.4, 4.27 and 1.04) and DAG (δ 2.36, δ 1.16 and δ 0.72). Severely overlapping signals were observed in the carbohydrate region (δ 3.0–5.5) (Fig. 2). Nevertheless, the anomeric proton of glucose could be observed at δ 5.16 and δ 4.6 while those of sucrose were detectable at δ 5.4, and δ 4.16. The signals of these compounds were detected in the ^1^H NMR spectra of both young and mature leaves. However, the signal intensities of the compounds were substantially higher for the young leaves. According to Mediani *et al*.^24^, the overall quality of a plant is influenced by variations in the metabolite profile over its growth periods. Thus, the metabolite variation of the young leaf samples was monitored at 14, 16, 18, 20 and 22 WAS towards determining to optimum harvest age of the herb.

**Figure 3:**
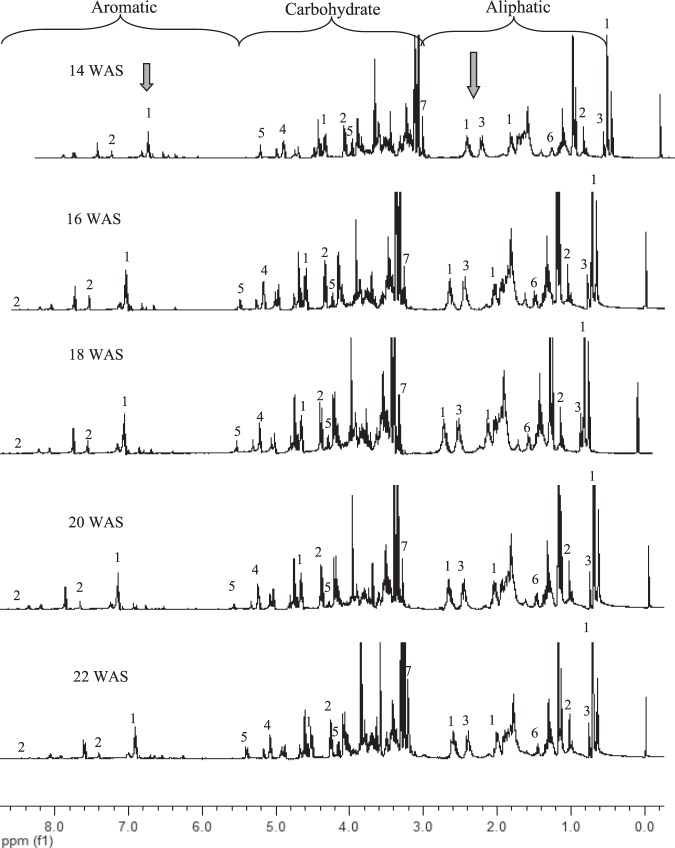
Full ^1^H NMR spectra of young leaves of *A. paniculata*, harvested at 14, 16, 18, 20 and 22 weeks after sowing (WAS) from δ 0.0 to 8.0 ppm. Andrographolide (1), neoandrographolide (2), and 14-deoxyandrographolide (3), glucose (4), sucrose (5), alanine (6) and choline (7).

The^1^H NMR spectra of young leaves harvested at 14, 16, 18, 20 and 22 WAS are shown in Fig. 3. The 14 and 16 WAS represented the herb’s vegetative stage, 18 WAS represented pre-flowering stage, while 20 and 22 WAS represented the flowering and seed formation stages, respectively (Fig. 1). As shown in Fig. 3, the representative ^1^H NMR spectra of the leaf extract at the various harvest age did not show visually significant differences between each other, except that, for leaf samples harvested at 16, 18 and 20 WAS, there was a noticeable increase in signal intensities for the peaks at δ 0.5–3.0, representing the signals for ANDRO and DAG. This is a visual indication that the levels of these two bioactive compounds are changing during the pre-flowering and flowering stages. Greater detail of the difference between young and mature leaves, as well as the changes observed over the different harvest ages^25,26^, were thus further evaluated via ^1^H NMR-based metabolomics analysis.

### Multivariate analysis of ^1^H NMR data

**Figure 4:**
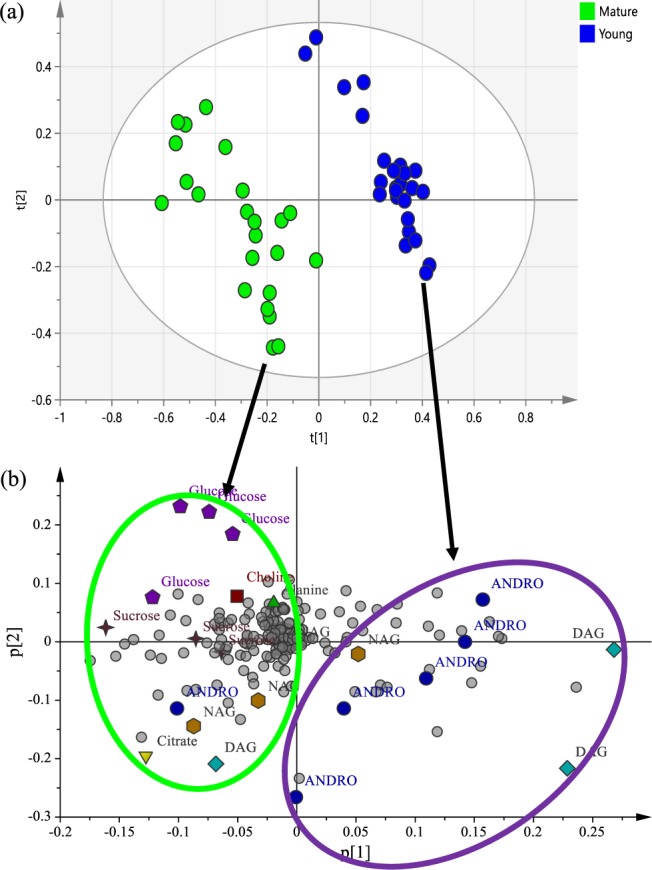
(**a**) PCA score plot for young versus mature leaves, along PC1 and PC2. (**b**) PCA loading plot showing the differentiating metabolites between young and mature leaves of *Andrographis paniculata*.

Principal Component Analysis (PCA) was performed to determine the difference between the leaf samples. The PCA score plot (Fig. 4a) showed clear cluster separation of the young (blue) and mature (green) leaves. All the samples in the score plot were within the 95% Hotelling T2 ellipse. The 2D plot showed that the best separation was obtained in PC 1 and PC 2 with an eigenvalue of 64%, the two PCs representing a variance of 18.7% and 45.8%, respectively. As shown in Fig. 4b, the PCA loading plot revealed that young leaves of *A. paniculata* have higher levels of the diterpene lactones, while mature leaves have higher levels of glucose (Fig. 4b). Most of the DAG signals also exist in the young leaves compared to the mature leaves. It is also noticeable that NAG was present in the young leaves. In contrast, the carbohydrate compounds and NAG as well as choline and alanine were dominant in the mature leaves (Fig. 4b).

**Figure 5:**
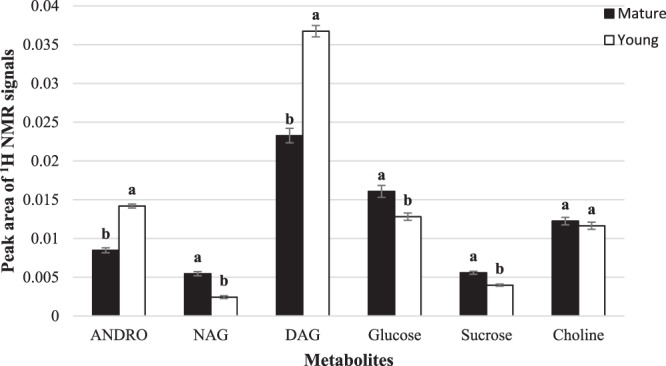
Peak area of ^1^H NMR signals of selected metabolites in young and mature leaves of *A. paniculata*. Mean values ± SE were from five replications. Different alphabets above each bar indicate significant differences at *P* ≤ 0.05 using the LSD test.

The levels of the discriminating metabolites were further analysed using one-way ANOVA. The characteristic signals of ANDRO, NAG, DAG, glucose, sucrose and choline were selected based on the most abundant signals relatively quantified by the ^1^H NMR spectra. Chemical shifts selected in the quantification were δ 4.52 (q) for ANDRO, δ 7.4 (s) for NAG, δ 0.72 (s) for DAG, δ 3.68 for glucose, δ 4.16 for sucrose and δ 3.2 for choline. Levels of ANDRO and DAG in the young leaves were significantly higher by 40% and 36%, respectively, compared to mature leaves. Conversely, the NAG level in young leaves was significantly lower than the mature leaves. Also, the levels of glucose and sucrose in the mature leaves were significantly higher by 20% and 28%, respectively, compared to the levels of glucose and sucrose in the young leaves (Fig. 5).

Other researchers have also reported the presence of higher levels of carbohydrate compounds in mature leaves. Shuib *et al*.^9^ reported similar results in that they found mature leaves to be richer in sugars and glycosidic compounds while the younger leaves have higher levels of the bioactive marker compounds and fatty acids^9^. Most studies have reported that the accumulation of secondary metabolites in young leaves is to protect against abiotic and biotic stress as well as to protect against insect pests and disease^9,27–29^. Brenes-Arguedas^28^ also stated that young leaves are more preferred by insect pest because the leaves are tender and nutritious. Also, the position of young leaves is more exposed to biotic and abiotic stress compared to the mature leaves^29^. Wingler *et al*.^30^ and Rolland *et al*.^31^ stated that sugar type compounds increased during senescence and accumulation of this compound lead to a repressed transcription of photosynthetic genes^32,33^. Shuib *et al*.^9^ reported that during leaf maturation, certain compounds might undergo enzymatic transformations or degraded to other secondary metabolites.

### Metabolite changes at different harvesting age in young leaves of Andrographis paniculata

**Figure 6:**
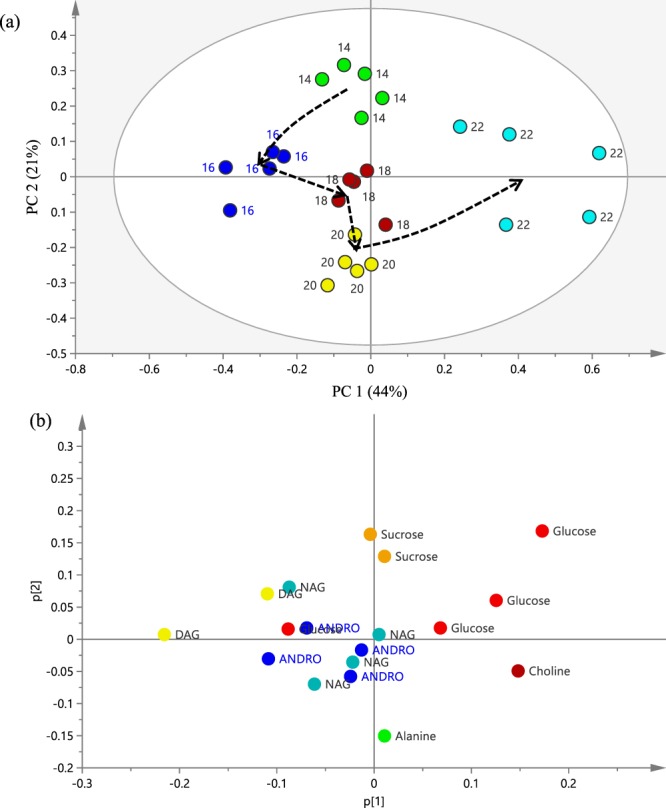
(**a**) Principal component analysis of young leaf samples harvested at 14, 16, 18, 20 and 22 WAS. Dashed arrow indicates the trajectory changes over the plant harvest ages. (**b**) The loading plots of metabolites for the first two principal components, PC1and PC2.

A PCA plot was further constructed for the ^1^H NMR data of young leaves at different harvest stages (Fig. 6a). In the model, 44% and 21% of the total variance could be explained by PC 1 and PC 2, respectively. The 22 WAS samples were distinctly separated from all the other samples by PC1, while the 14 WAS samples were separated from the other by PC2. Further examination of the loading plot (Fig. 6b) showed that the 22 WAS samples contained higher levels of glucose and choline, while the 14 WAS samples were richer in sucrose. Examination of the trajectory changes from the vegetative through to the post-flowering or seed formation stages, it could be observed from the loading plots that the levels of the diterpene lactones ANDRO, DAG and NAG were higher during the pre-flowering and flowering stages.

**Figure 7:**
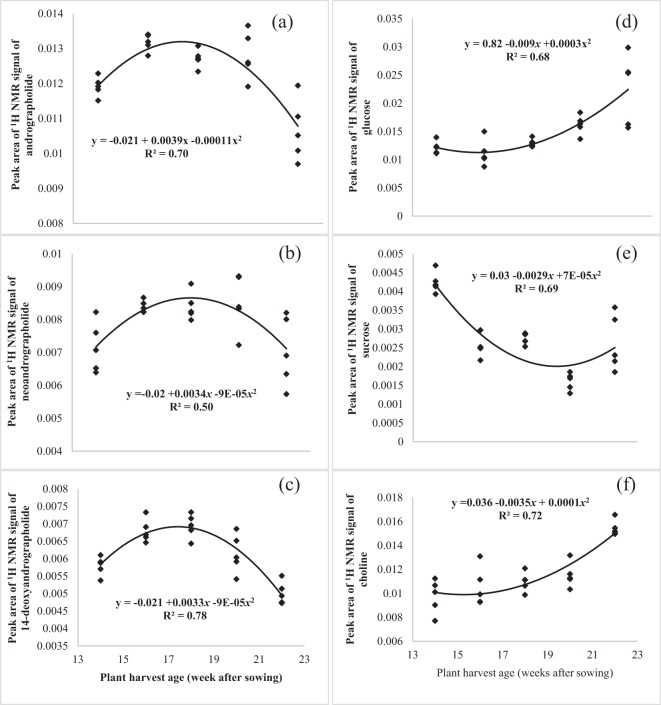
Relationships between plant harvest age (WAS) and peak area of ^1^H NMR signals of andrographolide (**a**), neoandrographolide (**b**), 14-deoxyandrographolide (**c**), glucose (**d**), sucrose (**e**), and choline (**f**). Solid line indicates a significant quadratic regression trend at *P* ≤ 0.05.

The levels of the discriminating metabolites were further analyzed by ANOVA and regression analysis. Chemical shifts selected in the quantification were δ 6.29 for ANDRO, δ 1.04 for NAG, δ 1.16 for DAG, δ 3.24 for glucose, δ 5.4 for sucrose and δ 3.2 for choline. From the results shown in Fig. 7, it can be seen that there was a significant quadratic relationship between harvest age and the levels of the diterpene lactones. The levels of ANDRO showed an increase from 14 WAS until 18 WAS, after which a pronounced decrease until 22 WAS (Fig. 7a). The quadratic equation explained that the optimum level peak area ^1^H NMR signals of ANDRO could be obtained 127 days after sowing, which coincided with the pre-flowering stage. A similar quadratic relationship with harvest age was observed for NAG and DAG (Fig. 7b,c). The optimum levels of NAG and DAG could be obtained 132 and 128 days after sowing, respectively, which were also within the pre-flowering stage.

The glucose and choline levels also showed significant positive and quadratic relationships with harvest age (Fig. 7d,f). Starting from 14 up to 22 WAS (the seed formation stage), the glucose and choline levels increased by 46% and 37%, respectively. Conversely, there was a significant, negative quadratic relationship between sucrose levels with plant harvest, with 72% (R^2^ = 0.72) of the variability being due to the plant harvest age. Sucrose levels showed a reduction from 14 up to 20 WAS. After that, however, the levels increased by 39% until it reached the seed formation stage (Fig. 7e).

The present study demonstrated how the metabolites of *A. paniculata* changed during its growth period. The results of the present study further reemphasise that the biosynthesis of certain plant secondary metabolites can increase or decrease with plant age^32^. Borzak *et al*.^33^ mentioned that the patterns of the metabolite changes in the plant varied from seedling to the mature stage due to the changes in the ontogenetic expression. Previous studies on Indian cultivars of *A. paniculata* also reported higher contents of the metabolites between 120 to 135 days after sowing^34^, 130 days after planting^35^ and 135 days after planting^36^. For the Malaysian cultivar selected for this study, the optimum time for harvest is therefore similar, falling well within the pre-flowering stage at 18 WAS. The lower levels of the metabolites at the seed formation stage could be explained by the occurrence of senescence of the older leaves^36^. According to Ainsworth and Bush^37^, the senescence pathway of older leaves started when the accumulation of carbohydrate in the older leaves resulted from the suppression of the photosynthetic gene expression in the older leaves. Therefore, the accumulation of glucose at 22 WAS could be attributed to the increase in total surface area of leaves, which indirectly increases the photosynthetic activity and the accumulation of sugar in the plant^11^.

## Conclusions

The present study used ^1^H NMR-based metabolomics combined with ANOVA and regression analysis to demonstrate the changes in the metabolite profile occurring in a local cultivar of *A. paniculata* during the period of its growth. Seven metabolites, including the bioactive compounds (ANDRO, NAG, and DAG), which were selected to represent quality parameter of the herb, were identified and their levels monitored in young and mature leaves, and then, over varying stages of its growth. Results from metabolomics and regression analysis revealed that young leaves of the herb are richer in the bioactive compounds, and the best time to harvest the herb is during the pre-flowering stage where the levels of these metabolites were observed to be at their highest. This information will be useful for farmers and entrepreneurs considering to venture into commercial herb farming where the objective is to produce superior and consistent quality of the plant material for further applications by the herbal, nutraceutical and pharmaceutical industries.

## Supplementary information

Supplementary File S1
