# Supplementary material for: Metabolite profiling of Andrographis paniculata (Burm. f.) Nees. young and mature leaves at different harvest ages using 1H NMR-based metabolomics approach
Source: Sci Rep. 2019 Nov 14;9:16766. doi: 10.1038/s41598-019-52905-z (PMC6856553; doi:10.1038/s41598-019-52905-z)
Supplement: Supplementary file 1 — Supplementary File S1 [file 41598_2019_52905_MOESM1_ESM.docx]

**Metabolite profiling of *Andrographis paniculata* (Burm. f.) Nees. young and mature leaves at different harvest ages using ^1^H NMR-based metabolomics approach**

Nor Elliza Tajidin^a,b^, Khozirah Shaari^c*^, Maulidiani Maulidiani^d^, Nor Shariah Salleh^b^, Bunga Raya Ketaren^b^, and Munirah Mohamad^b^

^a^ Faculty of Sustainable Agriculture, Universiti Malaysia Sabah, UMS Sandakan Campus, Locked Bag No. 3, 90509 Sandakan, Sabah, Malaysia.

^b^ Department of Crop Science, Faculty of Agriculture, Universiti Putra Malaysia, 43400 UPM, Serdang, Selangor, Malaysia.

^c^ Institute of Bioscience, Universiti Putra Malaysia, 43400 UPM Serdang, Selangor, Malaysia.

^d^School of Fundamental Science, Universiti Malaysia Terengganu, 21030 Kuala Terengganu, Malaysia

^*^ Corresponding Author: Khozirah Shaari

**Affiliation address:**

Institute of Bioscience,

Universiti Putra Malaysia,

43400 UPM Serdang, Selangor, Malaysia.

Telephone: 03-8946 8081

Fax: +603 8942 3552

Email: khozirah@upm.edu.my/ khozirah@yahoo.com.my

**Figure S1: ^1^H NMR spectrum (500MHz) of andrographolide (AG)**

**Figure S2: ^1^H NMR spectrum (500MHz) of 14-deoxyandrographolide (DAG)**

**Figure S3: ^1^H NMR spectrum (500MHz) of neoandrographolide (NAG)**

**Figure S4. 2D *J*-resolved spectra of the *Andrographis paniculata* extract in the region δ 0.0 to 8.8. The observed signals are as follows: andrographolide (1), neoandrographolide (2), and 14-deoxyandrographolide (3).**
